# It takes threat of Ebola to see lessons from low income countries

**DOI:** 10.1186/s12992-015-0102-3

**Published:** 2015-04-09

**Authors:** Matthew Harris, Viva Dadwal, Albert Wu, Shamsuzzoha B Syed

**Affiliations:** Commonwealth Fund Harkness Fellow in Healthcare Policy and Practice, New York University, New York, USA; Visiting Scholar, Johns Hopkins University Bloomberg School of Public Health, Baltimore, USA; Center for Health Services & Outcomes Research, Johns Hopkins University Bloomberg School of Public Health, Baltimore, USA; Johns Hopkins University Bloomberg School of Public Health, Baltimore, USA

In his recent perspective in the New England Journal of Medicine, the head of the United States (US) Centers for Disease Control and Prevention (CDC), Thomas Frieden, and colleagues, were concerned that the arrival of the Ebola virus (EVD) in Lagos, Nigeria heralded a concerning escalation of the epidemic in West Africa [1]. By 24th September 2014, a total of 20 EVD cases (19 laboratory confirmed, one probable) had been reported in Nigeria. All 20 cases stemmed from a single index patient, a traveller returning from Liberia on 20th July 2014. Unless Nigeria responded quickly and effectively, the virus would spread rapidly in Africa’s largest city and into the country beyond [2].

The subsequent successful containment of the virus in Nigeria led to a flurry of declarations in the media that much could be learned from Nigeria’s approach [3-6]. What was done right? Almost immediately after the first case was diagnosed, the Nigerian government declared a Public Health Emergency and centralised the command structure through an Ebola Response Incident Management Center [7]_._ Furthermore, they developed a robust and coherent Social Mobilization strategy that included multiple response teams spanning across several cities [7]. These teams conducted over 26, 000 rapid house-to-house, in-person visits to assess Ebola symptom development and ensure that community members understood risks and preventive steps [7-9]. Suspected patients were triaged and the areas they inhabited decontaminated [7]. Finally, to control and counter the outbreak of anxiety and hysteria triggered by early, inaccurate information [3,7], strong public awareness campaigns were deployed, making use of accurate and timely messages that were relayed not only by the national media [4], but also social media [8], Short Message Service (SMS) platforms [8], and traditional, religious and community leaders [4,6,9]. To sum it up, Nigeria’s outbreak response was vigorous, effective and coordinated.

Pleasing, though it is, to see successful experiences from an emerging economy given broad exposure, we believe that to fully embrace the potential learning two important issues ought to be addressed.

First, we must not assume automatically that the key features of the Nigerian response do not apply to more ‘advanced’ health systems like that of the US. Although the US boasts some of the world’s leading class hospitals, the healthcare delivery system here is also somewhat fragmented, with a primary care infrastructure that can be significantly strenthened [10]. This multiplies the likelihood for errors and system vulnerabilities. The handling of the first domestically diagnosed Ebola case in Dallas, Texas raised immediate concerns about US public health preparedness. Federal Public Health officials do not have the authority to command and control the kind of rapid, preventive and contact tracing activities deployed by Nigeria. Instead, the CDC must be expressly invited by a State government in order to effect public health responses at a local level. Perhaps as a result, we have seen everything from misdiagnoses, to delayed treatments, accidental in-hospital transmissions, and quarantine policy reversals regarding quarantine for healthcare workers returning from West Africa, and a regrettable politicisation of the public health response.

Second, it should not take an epidemic of unprecedented proportion, such as this Ebola crisis, to acknowledge that learning can run from the so-called “Global South” to the “Global North”. So deeply entrenched are the expectations that technical expertise flows only one way, that our mental blinders do not allow us to contemplate anything else in reverse. Sadly, this mental model has marked the global health dialogue on the subject of Ebola. The World Health Organization, the CDC, Médecins Sans Frontières (MSF), and UNICEF, all worked alongside the Nigerian government during the Ebola outbreak [7,9]. What were some of the lessons they learned and how can those lessons be adapted in other settings, including in the US? This should be a critical component of the development agenda, particularly as we move into intense dialogue around sustainable development goals in 2015. This journal’s dedicated open-access series on reverse innovation in global health systems aims to challenge conventional thinking by collecting evidence of bidirectional learning beyond the Ebola response [11,12]. Only by harvesting and actioning such questions will we be able to recognize the true potential of global innovation flow for health systems but perhaps beyond [13].

Since the outbreak began, there have been 24,247 reported Ebola cases and 9,961 reported deaths in nine countries [9]. On 20th October 2014, Nigeria was declared Ebola free [14] (WHO). Nigeria was able to win the battle against Ebola not only as a result of “doing the right things” but also because it drew on its strengths from past successful experiences, such as recent polio eradication campaigns [7]. With a dense population and overburdened infrastructure, Nigeria’s case prompts the recognition that preparedness depends on the core strengths of health systems. Indeed, increasing attention is now being placed by global partners and national authorities across Africa on health systems resilience for both Ebola affected countries and those seeking to bolster their abilities to respond to all potential hazards. For example, infection prevention and control – a core attribute of patent safety laid bare during the Ebola out break – requires strengthening of “everyday” health services. This needs to take into consideration issues related to workforce capacity, supplies of essential consumables, data & information, organization of health services along with quality improvement processes. Nothing comes easy in health systems strengthening but the gains are long term.

Clearly, not enough has been done to support well-functioning health systems in many African countries, but the US too is no exception to this rule. As each of the three high-transmission countries now moves forward on the immediate priority of getting to zero cases, a simultaneous emphasis is being placed on early recovery with a specific attention on the re-activation of essential health services. Multiple priorities emerge – immunization, malaria control, integrated management of childhood illness, maternal health to name a few. The challenge is to integrate these priority interventions within a comprehensive, people centred approach to re-designing service delivery with people and not diseases. Further, efforts to strengthen preparedness for future outbreaks needs to be integrated with the delivery of services using a “one health system” approach. Might there also be lessons that emerge from this upcoming effort here for the world? We believe that harvesting insights from these experiences will be important for the global pool of knowledge, for all countries.

Rare, novel infections like Ebola will continue to test our ability to respond to diseases. When the current crisis dies down, we might realise that strengthening health systems is actually a good plan for any disease or outbreak, not just for Ebola. At the same time we might also recognize that the fluid and rapidly evolving health systems in African countries may hold multiple ‘golden nuggets’ that can be utilized to strengthen systems in the US and elsewhere in the world. This requires a greater appreciation that examples of best practice can come from anywhere and flow anywhere, including from the “global south” to the “global north”.
